# Household presentation of acute gastroenteritis in a primary care sentinel network: retrospective database studies

**DOI:** 10.1186/s12889-020-08525-8

**Published:** 2020-04-05

**Authors:** Simon de Lusignan, Julian Sherlock, Filipa Ferreira, Sarah O’Brien, Mark Joy

**Affiliations:** 1 Nuffield Department of Primary Care Health Sciences, University of Oxford, Woodstock Rd, Oxford, UK; 2 Department of Clinical & Experimental Medicine, University of Surrey, The Leggett Building, Daphne Jackson Rd, Guildford, UK; 3 Royal College of General Practitioners Research and Surveillance Centre, 30 Euston Square, London, UK; 4 Department of Public Health and Policy, Institute of population Health Sciences, University of Liverpool, Liverpool, UK

**Keywords:** Gastroenteritis, Disease incidence, Infectious, Family characteristics, Population characteristics, Medical record systems, Computerised

## Abstract

**Background:**

Acute gastroenteritis (AGE) is a highly transmissible condition spreading rapidly between individuals and within households. Rotavirus vaccination was introduced in the UK in 2013. The study objectives were to investigate how acute gastroenteritis incidence changed over 25 years and household incidence of AGE since 2013.

**Methods:**

Repeated cross-sectional study of Royal College of General Practitioners Research and Surveillance Centre network. We used a negative binomial model to report incidence rate ratio (IRR) using the last 5 years data. We also conducted a retrospective cohort analysis, using a shared gamma frailty model (2013–2017). We explored the impact of child under 5- years, household size, socioeconomic status quintile, and rurality.

**Results:**

In the cross-sectional analysis, the IRR of AGE in households with a child of under 5 years was 12.20 (95%CI 11.08–13.45-, *p* < 0.001) compared with households without; the IRR fell across IMD quintiles, for example there is a 37% decrease in incidence comparing IMD quintile 1 to quintile 5 (95%CI -0.52-0.76, *p* < 0.001),

The cohort study revealed that the presence of an under 5 in the household was associated with a higher risk of household presentation (HR = 6.29, 95% CI 5.61–7.06, *p* < 0.001). In addition, we observe a reduction in risk of presentation from the most to the least deprived socioeconomic quintile (second quintile: HR = 0.74 (95%CI 0.59–0.92), to least deprived quintile, HR = 0.55 (95%CI 0.41–0.74). We saw a lower association with male gender, white ethnicity and living outside London, but an increased association with increasing household size.

**Conclusions:**

The incidence of AGE has changed over time: pre-school children, larger households, and living in London were associated with higher rates, and male gender and higher economic status associated with lower rates.

## Background

Acute gastroenteritis (AGE) contributes significantly to the burden of infectious diseases as well as having wider societal impact [1]. AGE is a readily transmissible condition spreading rapidly between individuals and within households [2, 3]. It is estimated that around 25% of the United Kingdom (UK) population suffers from an AGE episode per year. General practitioners only see the tip of the epidemiological iceberg, with around 2% of cases attending primary health care [4].

Children under 5- years are the most vulnerable group to develop AGE which is usually associated with rotavirus infection. Because rotavirus is the most prevalent cause, the World Health Organisation (WHO) recommends rotavirus vaccination for this age group [5]. Rotavirus vaccination was introduced in the UK in 2013 and produced a significant decrease in AGE presentation [6]. Despite this, much of the published research about AGE focusses on norovirus [7].

Whilst it is known that children aged under 5- years are important carriers of AGE [2, 8] and are more likely to spread it to older children and adults [9] we have been unable to identify any literature about the simultaneous presentation from the same household with gastroenteritis. We use the term “household incidence” to describe when two or more people from the same household present, as this may be due to either household transmission or common external exposure.

We carried out this study to describe household incidence of medically attended AGE. We report change in AGE incidence over the last 25 years, and investigated whether we could detect any change in household incidence in the last 5 years, since the introduction of rotavirus vaccine. We wished to describe, and if so to what extent the presence of children under 5 years old in a household are associated with a higher incidence of AGE.

## Methods

### Subjects and setting

We have published a detailed protocol for this study, it has two parts: a 25-year repeated cross-sectional study and a five-year retrospective studies of household incidence [10].

We used the Royal College of General Practitioners (RCGP) Research and Surveillance Centre (RSC) database, a well-established sentinel network database consisting of pseudonymised health care records of individuals in England to conduct this study [11]. The UK has a registration-based system in which one individual is registered with a single general practice (GP), giving a reliable denominator [12]. The database is one of the oldest sentinel networks [11] with practices receiving long-term feedback about data quality, most recently via a dashboard [13]. Gastroenteritis is one of the conditions monitored long-term, using a consistent definition [14].

We carried out a yearly cross-sectional analysis repeated over 25 years (1st January 1992 until 31st December 2017), reporting incidence over this period and the characteristics of this population. We also conducted two five-year studies of household incidence; we explored the incidence in the last 5 years in cross-sectional data and also conducted a retrospective cohort study (1st January 2012 to 31st December 2017). We restricted our exploration of household incidence to the last 5 years since the introduction of rotavirus vaccine in the UK [6] we also, only have reliable household data since 2013, this is also the time period.

### Trends in AGE over time and in household incidence

We used descriptive statistics to report any change in the population with AGE over the last 25 years, with stratification by age, gender, ethnicity and obesity, measured using body mass index (BMI). We used the World Health Organisation (WHO) classification of obesity in adults: Underweight (BMI < 18.5), normal (BMI 18.5–25), overweight (BMI 25–29.9), class 1 obesity (BMI 30–34.9), class 2 (BMI 35–39.9) and finally class 3 (BMI ≥40) [15]. All of these variables except obesity are presented for the whole population; the UK convention is not to calculate BMI from weight and height in children’s records [16].

### Definition and statistical analysis of household incidence

We defined a case of household incidence when two members of the same household presented on the same day or within 10 days. We identified cases of household incidence (*n* = 4346 for the cross-sectional study, and *n* = 3967 for the retrospective cohort) over the previous 5 years. We directly standardised AGE rates by age and gender, using the 2011 national census population [17], we reported rates per 100,000. Ethnicity recording was maximised using an ontological approach [18]. We used Index of Multiple Deprivation (IMD) quintile as a measure of deprivation, this is the National measure of small area socioeconomic status [19].

### Statistical methods repeated cross-sectional study

We employed a negative binomial model to study the potential impact of the presence of a child under 5 years old in a household on the incidence of gastro-enteritis. Data consisting of counts of transmissions of AGE in households was tested for over-dispersion (using the Cameron-Trivedi test [20], implemented in the R library AER, version 1.2–7). Evidence for over-dispersion was strong (*p* < 0.001) hence our adoption of the negative binomial model. We controlled for deprivation using the index of multiple deprivation (IMD) quintile, the urban-rural status of the household, their NHS Region and the cross-sectional year. We used Office of National Statistics (ONS) data, linked to post code, to group practices into conurbation, city and town, or rural; this is based on population density. We also looked for any north-south difference using English NHS Regions. These divide England into: north, midlands and east, south, and London. We fitted the negative binomial model using the MASS library in R, version 7.3–45 and report incidence rate ratios (IRRs) with 95% -confidence intervals.

We conducted a change point analysis, see supplementary file (S9 Table) under the strong assumption of at most one changepoint, we looked for any change in directly standardised rate that might have coincided with the introduction of rotavirus vaccine. We sought a breakpoint in the standardised rates of AGE from 2008 onwards, thereby excluding the rise in incidence prior to 2008. To reduce the detection of a possibly spurious change point we used a threshold for the test statistic equal to 3.45 (=1.5*log(10)) using the R-library changepoint, version 2.2.2.

### Variables

The response variable is household with two or more cases of AGE within 10 days, while the explanatory variables include the presence of a child under 5-years in household (a binary classification), IMD quintile (a 5 level categorical variable which measures socioeconomic status,quintile 1,most deprived to quintile 5, least deprived, reference quintile 1), Urban-Rural classification (a 3 level categorical variable including conurbation, rural and the reference; town and city), Ethnicity, a 5 level categorical variable consisting of Asian (reference), Black, Mixed, Other and White. NHS Region (a 4 level categorical variable including north, midlands and east, south, and the reference, London and cross sectional year.

### Statistical analysis retrospective cohort study

We employed a shared gamma frailty model with time-varying covariates to model gap times between incidence of AGE in household at the person level [21]. We used this model because over the 5 years of the longitudinal study, household incidence is a possibly recurrent event and the study population is clustered by household [22]. We controlled for potential confounding at the individual level due to sex, ethnicity, age band, deprivation, and at the household level for household size, urban-rural classification and NHS Region. The time varying covariates are: at the household level, size of household and the indicator for the presence of an under 5-year old in the household, at the person level age band. The frailty, random effects term is a continuous gamma distributed variable that describes excess risk or frailty for distinct households (due to correlations in event times), thereby addressing clustering at the household level [23]. We report the results as hazard ratios [24] together with 95% confidence intervals. We used the R package library frailtypack, v 1.3.5 [25].

We did not pursue the exploration of older children (over 5-years) because descriptive data suggested there was no difference in incidence. Our exploration dividing children into those under 2 years is included in the supporting information files (S6 Fig & S8 Table).

## Results

### 25-year repeated cross-sectional study (1992–2017)

After an early steady rise in both crude and standardised rates we observed a rise in the rate from 1992 through to the middle of the first decade of the new century then a major decline towards the end of our observation period. The later years appear to be clearly differentiated from the early years (Supplementary File S1 & S5 Tables, S1 & S5 Figs). Despite a recent decline incidence is still similar to that at the end of the start of the observation period.

**Fig. 1:**
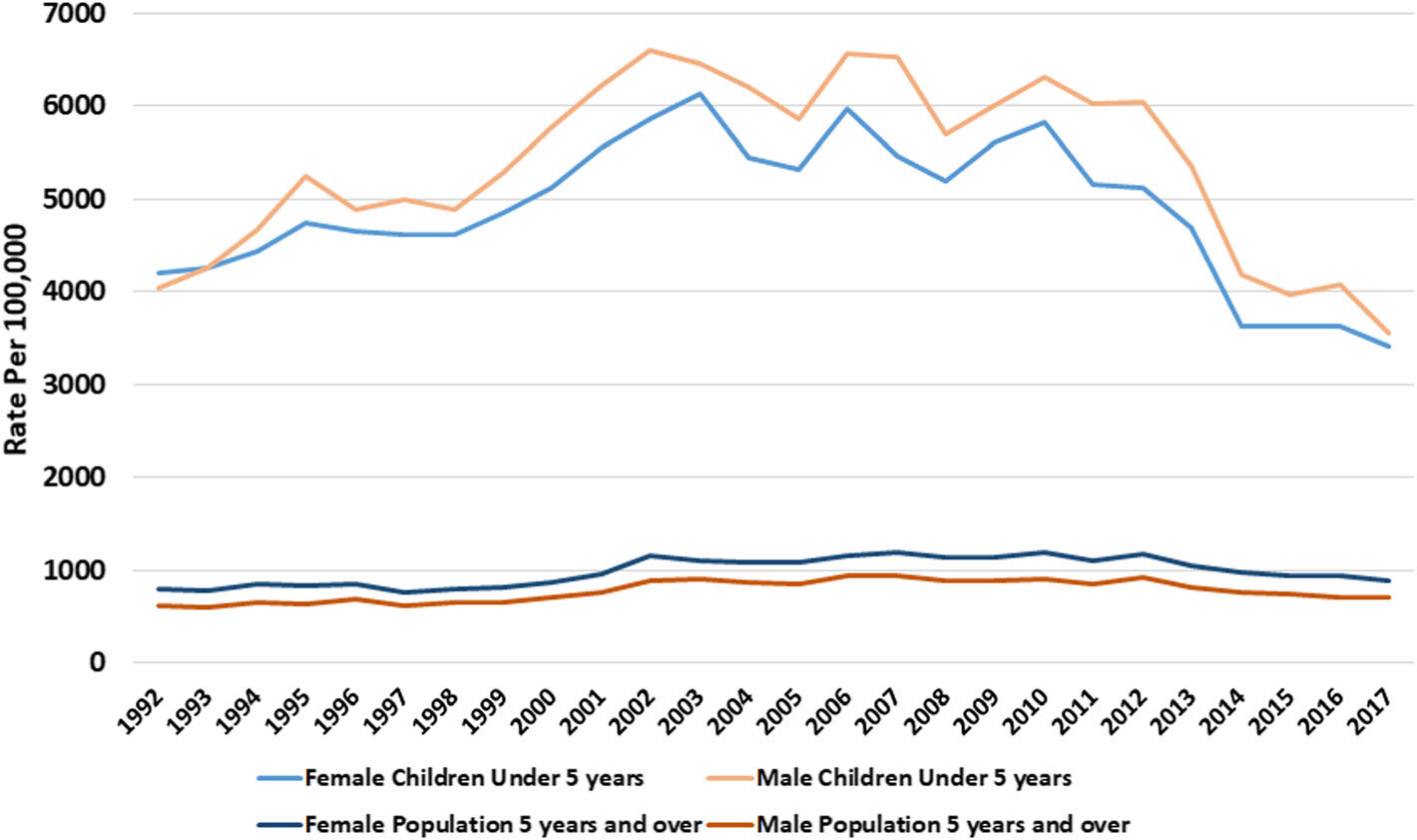
Acute gastroenteritis rates over 25 years, showing rates in boys and girls under 5 years old and males and females 5 years and over

We noticed differences in AGE between genders, ethnicities, and BMI. AGE incidence in the 0–4-year age band was on average approximately five times than in all other age bands; a rate of 5128 cases per 100,000 registered (95%CI 4975–5281), compared with 919/100,000 registered (95%CI 902–935), respectively (Fig. 1, S2, S3 & S10 Tables). Over the full 25-year period AGE presented more in boys 0–4 years old. There were no gender differences between 5 and 17 years, and in all other age bands females presented more than males (S2, S3 and S4 Figs).

**Fig. 2:**
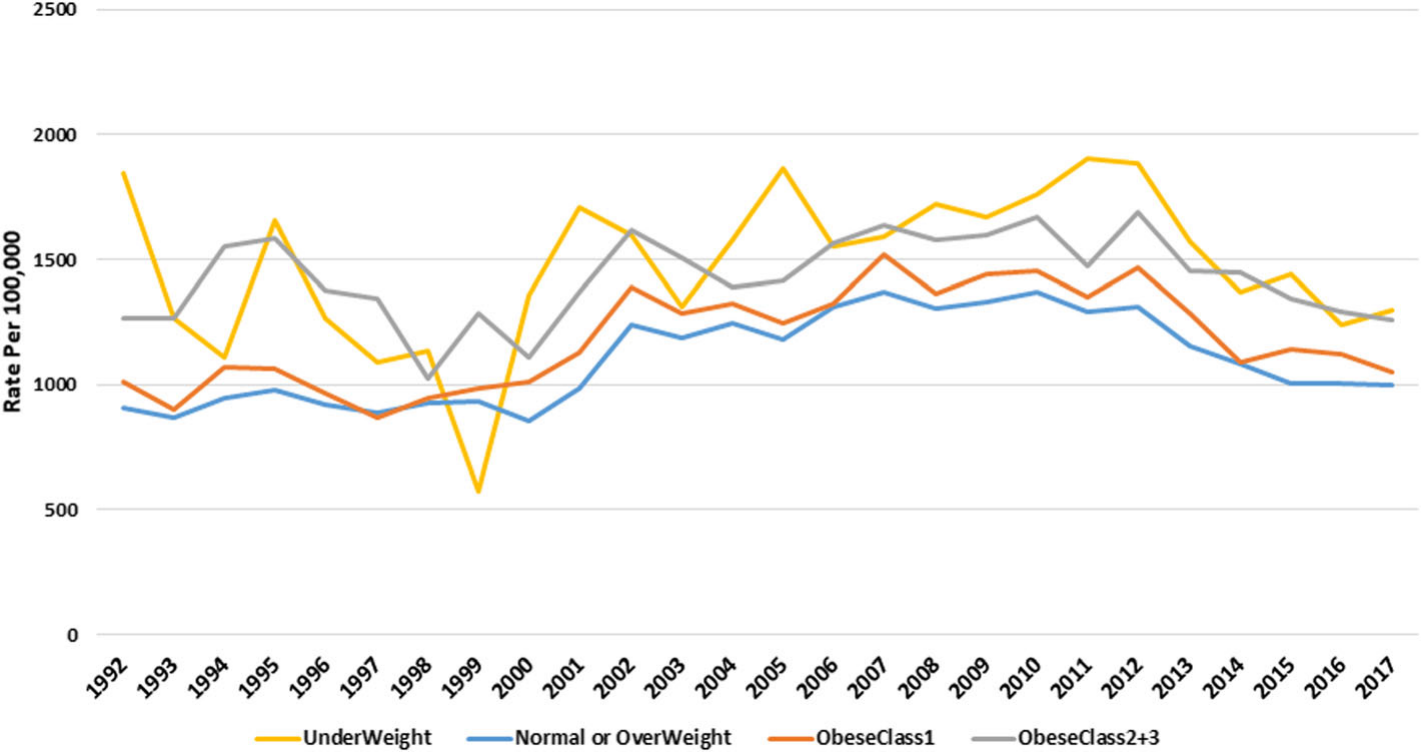
Acute gastroenteritis rates, in adults by combined WHO obesity category. Normal and overweight categories, and Class 2 and 3 obesity are combined

AGE rates were generally higher with increasing obesity except for underweight adults (BMI < 18.5 kg/m^2^) who presented more. Underweight adults had the highest rate of gastroenteritis, though it fluctuated over time. (Fig. 2, S4 & S11 Table). The underweight presented at similar rates to those with Class 2 and Class 3 obesity. The mean rate of AGE over the 25- year period was 1187 (95%CI 1120–1256) per 100,000 for adults with a normal BMI, 1139 (95%CI 1070–1210) for overweight adults, 1339 (95%CI 1256–1423) for obese adults, and 1535 (95%CI 1240–1845) for underweight adults.

### Five-year repeated cross-sectional study household incidence

**Fig. 3:**
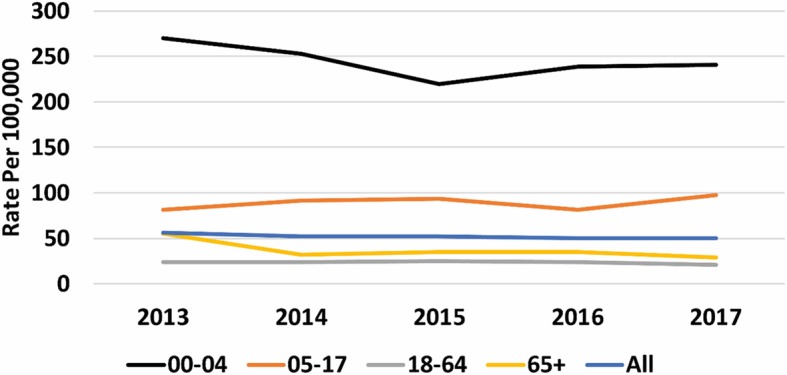
Rates of household incidence by age-band. The figure displays the rate of household incidence of AGE, per 10,000 registered patients by age band; 0–4 years have the highest rate compared to other age-bands across all 5 years

Generally, the pattern of household incidence reflected that seen in the descriptive analysis of AGE rates. Rates were much higher in children under 5-years compared with other age groups (Fig. 3, S12 Table). The rates on average were 230 per 100,000 (95%CI: 203–257) and 30 per 100,000 (95%CI: 27–32) for under 5- years and those 5- years or older respectively. We also reported rates in children under 2 years old (S6 & S8 Figs, S8 Table,). There were no clear differences between the ethnic groups over the twenty-five years observed, but in the last 5 years Asian, Black, mixed and other had higher rates while white ethnicity had lower rates (S7 Fig, S6 & S7 Tables).

**Fig. 4:**
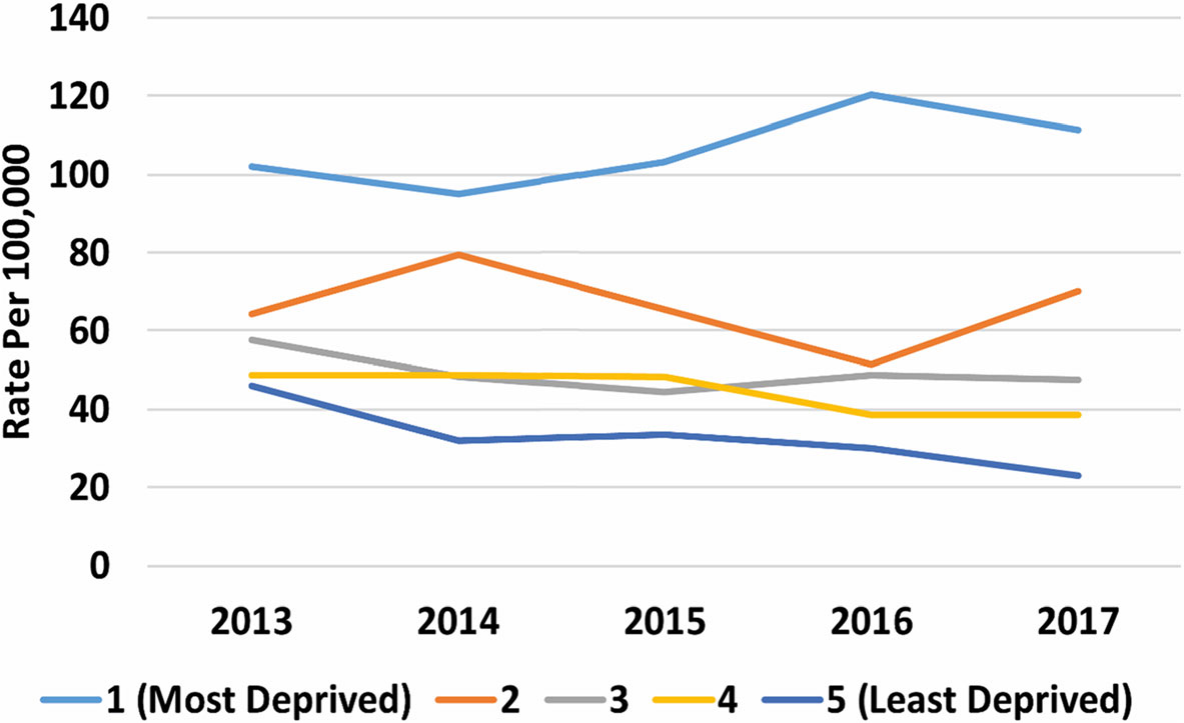
Rate of household incidence by socioeconomic status reported in IMD quintiles (1 is most deprived, 5 is least deprived)

**Fig. 5:**
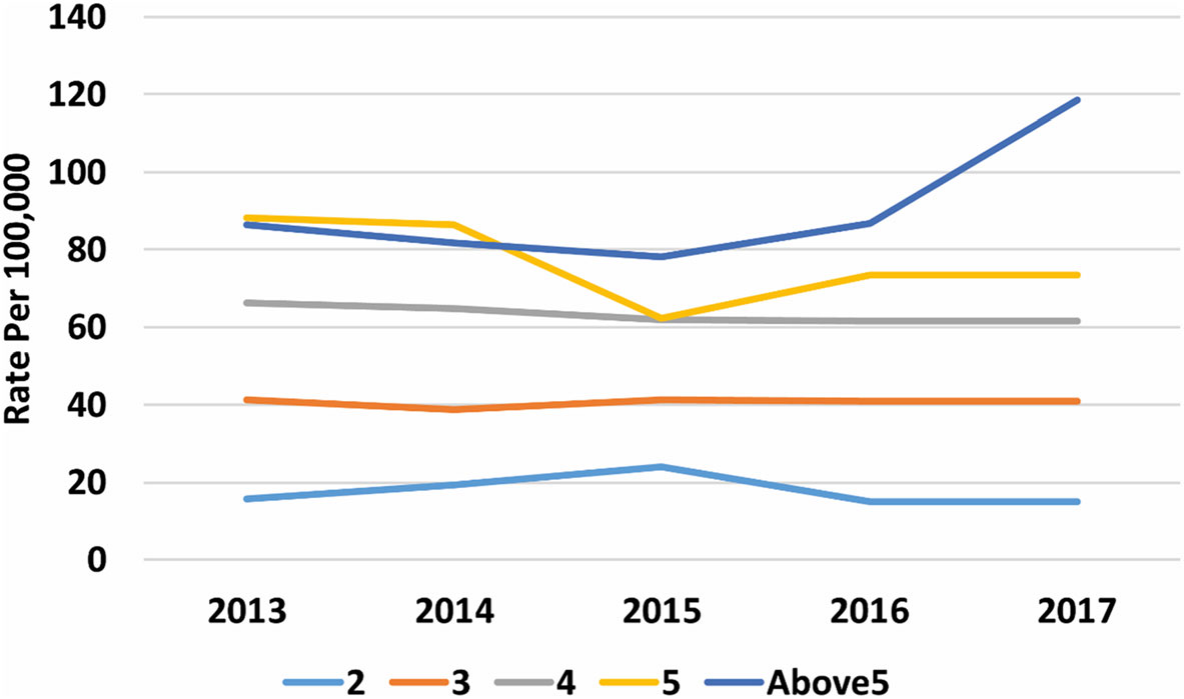
Household incidence of AGE by household size

**Fig. 6:**
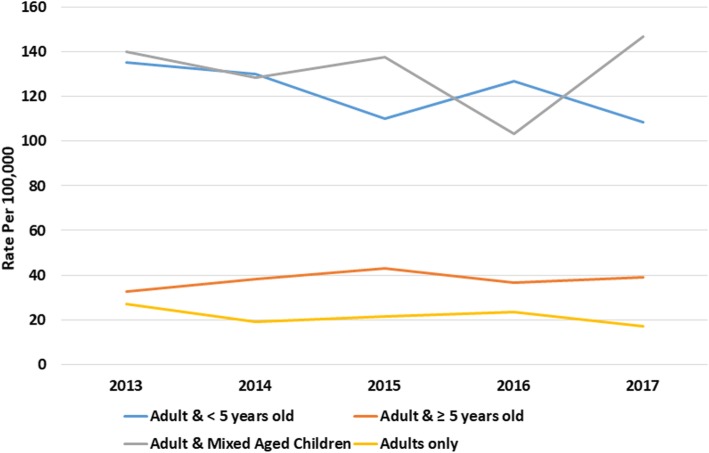
Household incidence of AGE by household type

**Fig. 7:**
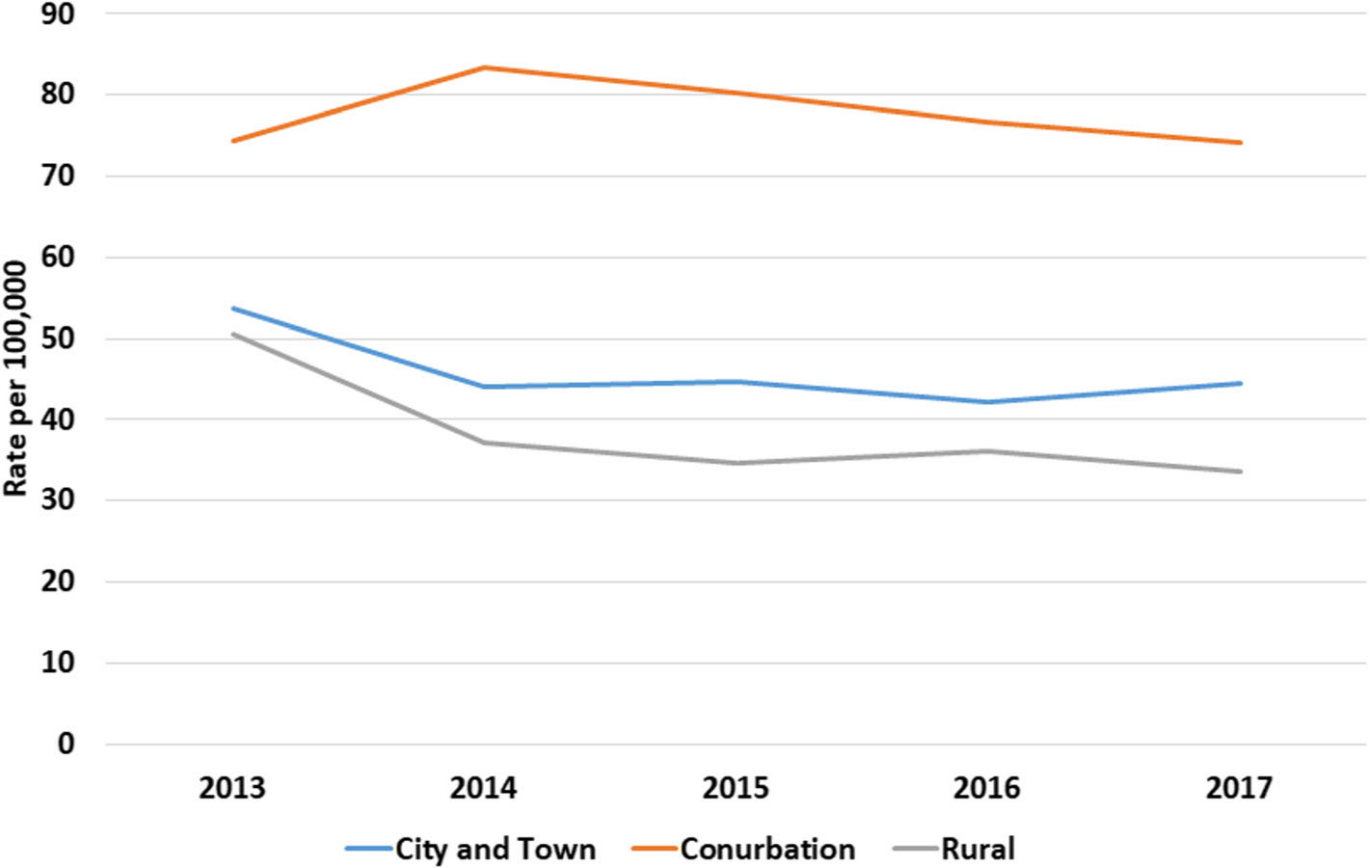
Household incidence of AGE by urban-rural classification

**Fig. 8:**
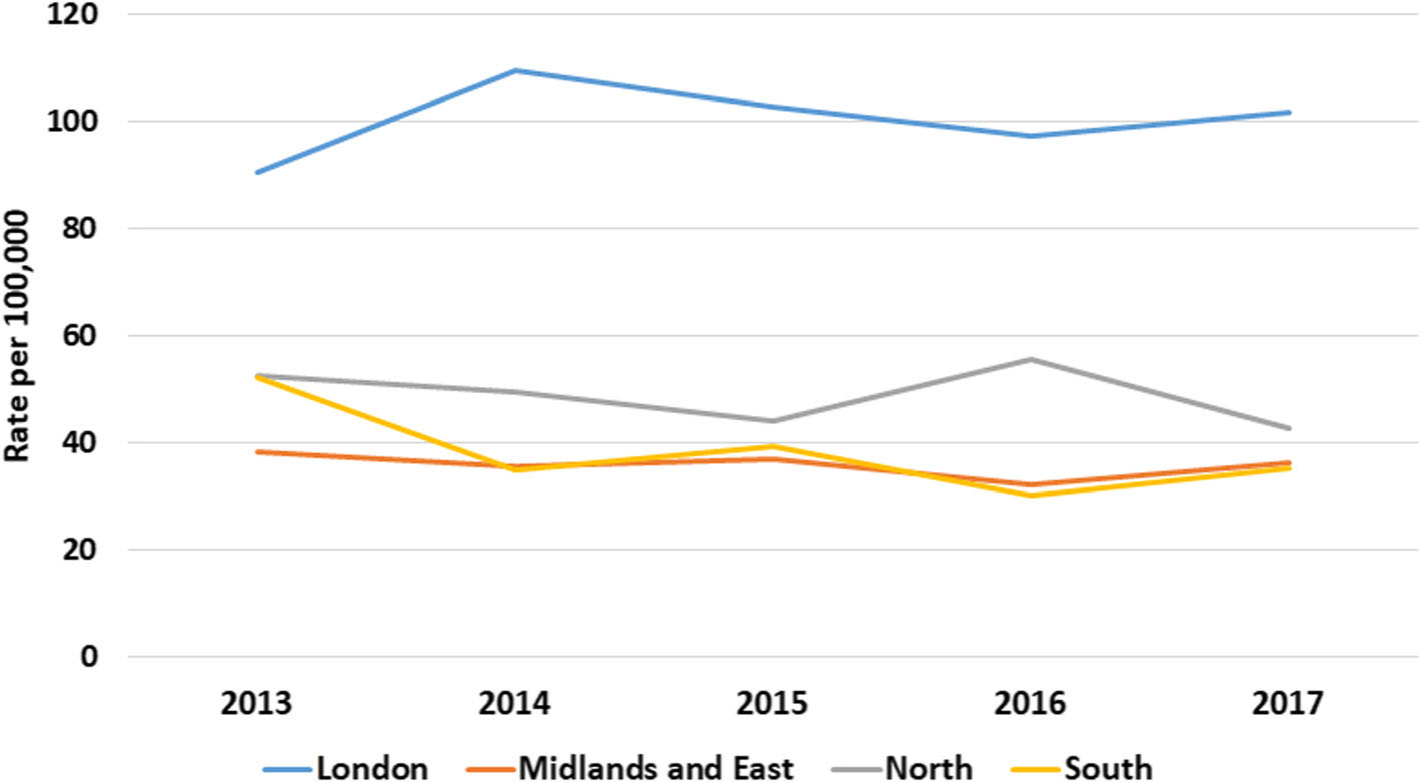
Household incidence of AGE by NHS Region

There was a clear gradient in socioeconomic status in the household incidence of AGE, with the most deprived having higher rates and the least deprived the lowest (Fig. 4, S13 Table). Similarly, incidence of AGE rose with increasing household size (Fig. 5, S14 Table). Household composition also made a difference, with households with children just under 5-years and children both under and over 5-years old had higher rates of household incidence (Fig. 6, S15 Table), as did living in a conurbation (Fig. 7, S16 Table) and NHS Region (Fig. 8).

### 5 year’s cross-sectional data study of household incidence (2013–2017)

In the 5 years data (2013–2017), there were 4346 incidence cases. We report the IRR from our negative binomial model (Table 1). A child under 5-years old in the household leads to an over 12-fold (12.20, 95% CI 11.08–13.44) increase in the expected count of events (*p* < 0.001), compared to a household without an under 5-years old resident. Compared to the most deprived quintile other quintiles show a reduction in incidence (e.g. the least deprived Quintile 5 shows a reduction in incidence of about 27% (IRR: 0.63, 95% CI 0.52–0.76). Compared with the reference category City and town (medium density suburban housing), conurbations (highest population density) showed a decrease in incidence by 24%. A rural setting did not show any difference. All the other NHS Regions had lower rates of household incidence, around half that of London (*p* < 0.0001). The observed fall per year in incidence rates was not statistically significant.

**Table 1:** Results of the negative binomial model of the 5 years cross-sectional data

Variable	Reference/Comparison	IRR	95% CI	*p*-value
< 5 years old in household	No < 5 years old in household	12.4	(11.3–13.7)	< 0.001
IMD Quintile	IMDQ1			
IMDQ2		0.73	(0.59–0.89)	< 0.001
IMDQ3		0.66	(0.53–0.82)	< 0.001
IMDQ4		0.76	(0.62–0.94)	0.01
IMDQ5		0.68	(0.55–0.84)	< 0.001
Ethnicity	Asian			
Black		0.51	(0.35–0.75)	< 0.001
Mixed		0.69	(0.42–1.13)	0.14
Other		1.03	(0.59–1.79)	0.93
White		0.64	(0.5–0.81)	< 0.001
Urban Rural Classification	Town & City (suburban)			
Conurbation		0.76	(0.61–0.97)	0.03
Rural		1.17	(0.95–1.43)	0.13
NHS Region	London			
Midlands & East		0.47	(0.36–0.63)	< 0.001
North		0.50	(0.40–0.63)	< 0.001
South		0.60	(0.46–0.80)	< 0.001
Year		0.96	(0.92–1.0)	0.2

Table 1 describes findings from the negative binomial model of the cross- sectional data. In the 5 years (2013–2017), there were 4346 incidence cases of AGE. The presence of a child under 5-years old in the household leads to an over 12-fold (12.20, 95% CI 11.08–13.44) increase in the expected count of events (*p* < 0.001), compared to a household without an under 5-year old resident. Compared to the most deprived quintile other quintiles show a reduction in incidence (e.g. the least deprived Quintile 5 shows a reduction in incidence of about 27% (IRR: 0.63, 95% CI 0.52–0.76). Compared with the reference category City and town (medium density suburban housing), conurbations (highest population density) showed a decrease in incidence by 24%. A rural setting did not show any difference. All the other NHS Regions had lower rates of household incidence, around half that of London (*p* < 0.0001). The observed fall per year in incidence rates was not statistically significant

*IRR* incidence rate ratio, *CI* confidence interval, *IMD* index of multiple deprivation

### 5-year retrospective cohort study (2013–2017)

There were 2,583,697 individuals in 1,150,278 households in our retrospective cohort study. Within this group were 3967 cases of AGE household incidence (members of the cohort had to be registered on the study index date). Approximately 27% of households with two or more people had a child under 5 years (though this varies from year to year). The median household size was 3.0 (interquartile range 2.0, maximum 11, mean 3.4). We also included gender, ethnicity, IMD urban-rural classification, and NHS Region in our model (Table 2).

**Table 2:** Demographic characteristics of individuals and households: 5 years retrospective cohort study

**Category**	**Variable**	**N**	**%**
**Gender**	*Female*	1,325,157	51.3
	*Male*	1,258,540	48.7
**Ethnicity**	*White*	1,622,038	62.8
	*Asian*	172,388	6.7
	*Black*	90,790	3.5
	*Other*	25,320	0.98
	*Mixed*	50,162	1.94
	*Unknown*	622,999	24.1
**IMD Quintile**	*1 (Most deprived)*	454,849	17.6
	*2*	470,726	18.2
	*3*	475,616	18.4
	*4*	542,786	21.0
	*5 (least deprived)*	639,720	24.8
**Urban Rural**	*City and Town*	518,210	45.1
	*Conurbation*	450,528	39.2
	*Rural*	181,540	15.8
**NHS Region**	*London*	257,768	22.4
	*Midlands and East*	191,617	16.7
	*North*	349,583	30.4
	*South*	351,647	30.6
**Total**		**2,583,697**	**100**

There were 2,583,697 individuals in 1,150,278 households in our retrospective cohort study. Within this group were 3967 cases of AGE household incidence. Approximately 27% of households with two or more people had a child under 5 years (though this varies from year to year). The median household size was 3.0 (interquartile range 2.0, maximum 11, mean 3.4)

The results of the frailty model show that there is an increased hazard ratio of household incidence where there is a child under 5-years old in a household (HR = 6.29, 95%CI 5.61–7.06). There is a decreasing trend in hazard ratios for IMD quintiles from most deprived category to least deprived: 0.74 (95%CI 0.59–0.92) for the second more deprived quintile through to 0.55 (95%CI 0.41–0.74) for the least deprived quintile. Overall, male gender was associated with less AGE (HR 0.98, 95%CI 0.88–1.08). White ethnicity (HR 0.69, 95%CI 0.54–0.88) had a lower HR than Asian but there were no other statistically significant differences. Each additional member of a household increased the HR of household incidence (HR 1.31, 95%CI 1.26–1.36). Conurbations had less household incidence than city and town, but there were no differences in rural settings. London NHS Region had a greater hazard of AGE than other parts of the country (Table 3); this difference is almost double.

**Table 3:** Results of the frailty model, showing the variables included, reference group and the hazard ratio (HR) of household incidence of acute gastroenteritis

Variable	Reference/Comparison	HR	95% CI	p-val
< 5 years old in household	No < 5 years old in household	6.29	(5.61–7.06)	< 0.001
**For each increase in household size**	Each additional person	1.31	(1.26–1.36)	< 0.001
IMD Quintile	IMDQ1			
IMDQ2		0.74	(0.59–0.92)	0.01
IMDQ3		0.70	(0.54–0.90)	0.01
IMDQ4		0.59	(0.46–0.75)	< 0.001
IMDQ5		0.55	(0.41–0.74)	< 0.001
**Gender**	Female	0.98	(0.88–1.08)	0.67
Ethnicity	Asian ethnicity			
*Black*		0.99	0.76–1.28	0.91
*Mixed*		1.04	0.74–1.44	0.84
*Other*		1.03	0.70–1.50	0.89
*White*		0.69	0.54–0.88	< 0.001
Urban Rural Classification	Town & City (suburban)			
Conurbation		0.80	(0.52–1.23)	0.31
Rural		1.06	(0.80–1.39)	0.69
NHS Region	London			
Midlands & East		0.43	(0.33–0.57)	< 0.001
North		0.64	(0.52–0.80)	< 0.001
South		0.53	(0.40–0.70)	< 0.001

Table 3 shows the results of the frailty model. We found that there is an increased hazard ratio of household incidence where there is a child under 5 years old in a household (HR = 6.29, 95%CI 5.61–7.06). There is a decreasing trend in hazard ratios for IMD quintiles from most deprived category to least deprived: 0.74 (95%CI 0.59–0.92) for the second more deprived quintile through to 0.55 (95%CI 0.41–0.74) for the least deprived quintile. Overall, male gender was associated with less AGE (HR 0.98, 95%CI 0.88–1.08). White ethnicity (HR 0.69, 95%CI 0.54–0.88) had a lower HR than Asian but there were no other statistically significant differences. Each additional member of a household increased the HR of household incidence (HR 1.31, 95%CI 1.26–1.36). Conurbations had less household incidence than city and town, but there were no differences in rural settings. London NHS Region had a greater hazard of AGE than other parts of the country

*HR* hazard ratio, *CI* confidence interval, *IMD* index of multiple deprivation

## Discussion

### Principal findings

The frailty survival analysis showed that children under 5-years living in a household increased the rate of household incidence of AGE. This finding fits with our cross-sectional and descriptive observations. We have also seen a rise then fall in the incidence of AGE in those under 5-years old over the period of the study, with rates of disease back at the level they were at the start of the observation period. Other factors associated with increased incidence were female gender, lower socioeconomic status, Asian ethnicity compared with white, and living in town and cities. The NHS London region was associated with an increased hazard ratio. The cross-sectional count model produced similar findings.

Presentation with AGE is around four times greater in under 5-year olds than other age-groups. Boys under 5-years present more than girls. In over 15-years of age, females present more than males. Lower socio-economic status and larger household size are both associated with an increased incidence. People who are underweight and those with class 2 or 3 obesity are diagnosed as having AGE more commonly than other body weights. This pattern has been reported by another study, though in a population of older hospitalised patient [26].

### Implications of the findings

Household incidence remains an important factor in the spread of AGE, and we have quantified the increased hazard based on cases reported to a sentinel network. To the best of our knowledge this is the first study to report household incidence of AGE based on direct observation of routine data.

The factors associated with increased household incidence may provide some insights into where public health interventions might best be directed. Gender differences are hard to explain, but we have reported, by way of contrast, greater presentation of boys with respiratory infections and atopic conditions to primary care [27]. We are unclear as to whether this is related to disease frequency or propensity to consult, of if due to another cause of gender bias. Deprivation may be associated with greater housing density [28] and this could be postulated for the higher rate in London in particular.

It is possible that underweight adults presenting with AGE may have other diagnoses that have not been recognised. We are unclear why obese patients presented more with AGE; it is possible that there may be a dose-related increased risk of AGE with increased food consumption.

Notwithstanding the successful introduction of rotavirus vaccine household incidence remains an issue. Rates of AGE are similar to those at the end of the last century and are due to infections other than rotavirus [29]. It is also possible that other public health measures and guidance account for the possible fall in AGE incidence, for example National Institute for Health and Care Excellence (NICE) guidance on AGE was published in 2009 [30].

We are unclear why household rates rose during the 1990s and first decade of the new century. A possible explanatory factor was the much greater promotion of pre-school education in England. In 1996 the government launched a nursery voucher scheme, spreading out nationally in 1997. Free entitlement for 3 -year olds rose from 37% in 1999 to 88% in 2007 [31]. But in 2010 the proportion of 3-year olds in nursery education was 92% and for 4-year olds 98% [32]. The main rise in AGE in children under 5-years follows the start of the nursery voucher scheme. Sometimes pre-school nurseries insist on GP review before allowing children back.

### Comparison with the literature

Previous studies have found evidence of household spread but used different approaches. Three studies looked at presentations with AGE and followed up the index case for evidence of household incidence. The first of these took the index case as the first presenting, and found that the younger the index case, the higher the odds of a further case. The OR of spread was 15.4 when the index case was 0-14 yr, 0.3 when 15–59 years old and 0.6 when 60 years or older. They also found higher OR with increasing household size: OR: 2.3 and 1.7 when households have five and six members, respectively [33].

A second study followed up households of people presenting with AGE. For a child under 2 years old the OR of acquiring AGE from an index case was 8.0; the OR for 2–5 year olds was 3.0, and 2.0 for those aged 6–17 years [34]. A final study enrolled adult index cases with AGE and looked for household spread. If the household had a child aged 2 years or younger the OR of incident was 2.57 [35]. A study of norovirus spread did not confirm these findings [36]. However, a further study on rotavirus, which focussed on vomiting as being important in incidence, showed greater odds of spread when vomiting was present (OR 11.6), as well as when children are younger (OR 48 for children 6–17 months compared to those > 18 months) [37].

Other public health interventions may have impacted on AGE rates over the period of this study. Changes over this period include encouraging handwashing [38], avoiding washing of meat and poultry and other measures to prevent cross-contamination of kitchens [39, 40]. However, reports from recent surveys suggest there are variable rates of uptake [41, 42].

### Limitations of the study

There are limitations to any study based on routine data. However, the RCGP RSC network improved its reporting of AGE through the development of an ontology to maximise case finding [9].

This study only looked at medically attended cases and there are likely to be many more. Whilst we looked descriptively at these data and ran a negative binomial model analysis of the cross-sectional data, our strongest findings are from the frailty model.

There are limitations to our study, related to the definition of transmission and data collection method. We can’t differentiate what might be increased propensity to consult, or to attend with an unwell child under 5-years than an older adult, something demonstrated in rotavirus infection [43]. However, the propensity to consult is also impacted by gender, ethnicity or socioeconomic status [27]. However, the need to consult is also driven by the health of the child and our pattern of attendance appears to be similar to that of admission to hospital with AGE [44, 45].

We only reported the cases of household incidence in the last 5 years (*n* = 4345) because our system of household linkage was not in place prior to this, though this conveniently fitted with the period after the introduction of rota virus vaccine. If we could have assumed that households had not changed in the previous 20 years, we could have identified more cases (*n* = 16,832). We felt it was unsafe to include these in our analysis. Whilst this is a limitation, it also demonstrates that household incidence continues notwithstanding the introduction of rotavirus vaccine [6].

Whilst over the 25-year observational period the rate of AGE increased (consultations rose from 4.67 per person per year in 2007–08, to 5.16 in 2013–14), it is possible that the propensity to consult and the threshold for presentation may have changed over this period [46]. We only looked for main effects, we did not look for interaction and possible confounders. For example, it is known that household size may be related to poverty, it is considered that this effect had been reduced over time [47, 48].

### Call for further research

This study suggests it is feasible to detect household incidence in a sentinel network, using routine primary care data. Household incidence of other infections should also be quantified, particularly influenza where the introduction of childhood vaccine aims to target the carriers the disease.

Research is also needed to explain why underweight adults more frequently present with AGE. They should possibly be tested for malabsorption and/or intolerances.

## Conclusions

Household incidence of gastroenteritis can be detected from routine data collected within a sentinel network. Household incidence of AGE continues, notwithstanding the introduction of rotavirus vaccine. Pre-school children, larger households, city and town location (medium density housing), and London were associated with higher risk. Male gender, higher economic status, and conurbations outside London were associated with lower rates. Further public health interventions are required to reduce the spread and incidence of AGE.

## Supplementary information

**Additional file 1: Figure S1.** Incidence of gastroenteritis (age and gender standardised rate per 100,000 registered) in RCGP RSC sentinel practices 1992-2017 (standardised against the 2011 Census).

**Additional file 2: Figure S2.** Crude gastroenteritis rates age band 05-17years.

**Additional file 3: Figure S3.** Crude gastroenteritis rates age band 18-64years.

**Additional file 4: Figure S4.** Crude gastroenteritis rates age band 65 and above.

**Additional file 5: Figure S5.** Crude gastroenteritis rates age band 00- 4years.

**Additional file 6: Figure S6.** Gastro incidence rates per 1000 total by age bands.

**Additional file 7: Figure S7.** Crude gastroenteritis rates by ethnicity.

**Additional file 8: Figure S8.** Household incidence gastroenteritis ethnicity rates.

**Additional file 9: Table S1.** 2011 census data and proportion.

**Additional file 10: Table S2.** Age and sex standardised rates.

**Additional file 11: Table S3.** Gastroenteritis rates.

**Additional file 12: Table S4.** Gastroenteritis rates with confidence interval.

**Additional file 13: Table S5.** Title: Gastroenteritis rates age and sex.

**Additional file 14: Table S6.** Crude gastroenteritis rates by ethnicity.

**Additional file 15: Table S7.** Ethnicity.

**Additional file 16: Table S8.** Gastroenteritis rates by age band including under 2 years old.

**Additional file 17: Table S9.** Variance inflationary factors associated with the Frailty Survival Model.

**Additional file 18: Table S10.** Acute gastroenteritis rates over 25 years, showing rates in boys and girls under five years old and males and females five years and over. **Table S11.** Acute gastroenteritis rates, in adults by combined WHO obesity category. **Table S12.** Rates of household incidence by age-band. **Table S13.** Rate of household incidence by socioeconomic status reported in IMD. **Table S14.** Household incidence of AGE by household size. **Table S15.** Household incidence of AGE by household type. **Table S16.** Household incidence of AGE by urban-rural classification.

## Data Availability

Data supporting study findings are held in the secure server at the University of Surrey. RCGP RSC patient level data are available to researchers but not to the public. Details about how to request access to the data is given online at: www.rcgp.org.uk/rsc. Such approval is subject to the appropriate level of ethical approval and granted by the RCGP Data Access Committee on a project-by project basis. The RCGP has a permissive approach to additional research and studies to validate our findings.

## Abbreviations

AGE: Acute gastroenteritis
BMI: Body Mass Index
GP: General Practice
HR: Hazard ratio
IMD: Index of multiple deprivation
IRR: Incident rate ratio
NHS: National Health Service
NICE: National institute for Health and Care Excellence
ONS: Office of National statistics
OR: Odds ratio
RCGP: Royal College of General Practitioners
RSC: Research and Surveillance Centre
UK: United Kingdom
WHO: World Health Organisation
