# Supplementary figures and images for: Household presentation of acute gastroenteritis in a primary care sentinel network: retrospective database studies
Source: BMC Public Health. 2020 Apr 5;20:445. doi: 10.1186/s12889-020-08525-8 (PMC7132989; doi:10.1186/s12889-020-08525-8)

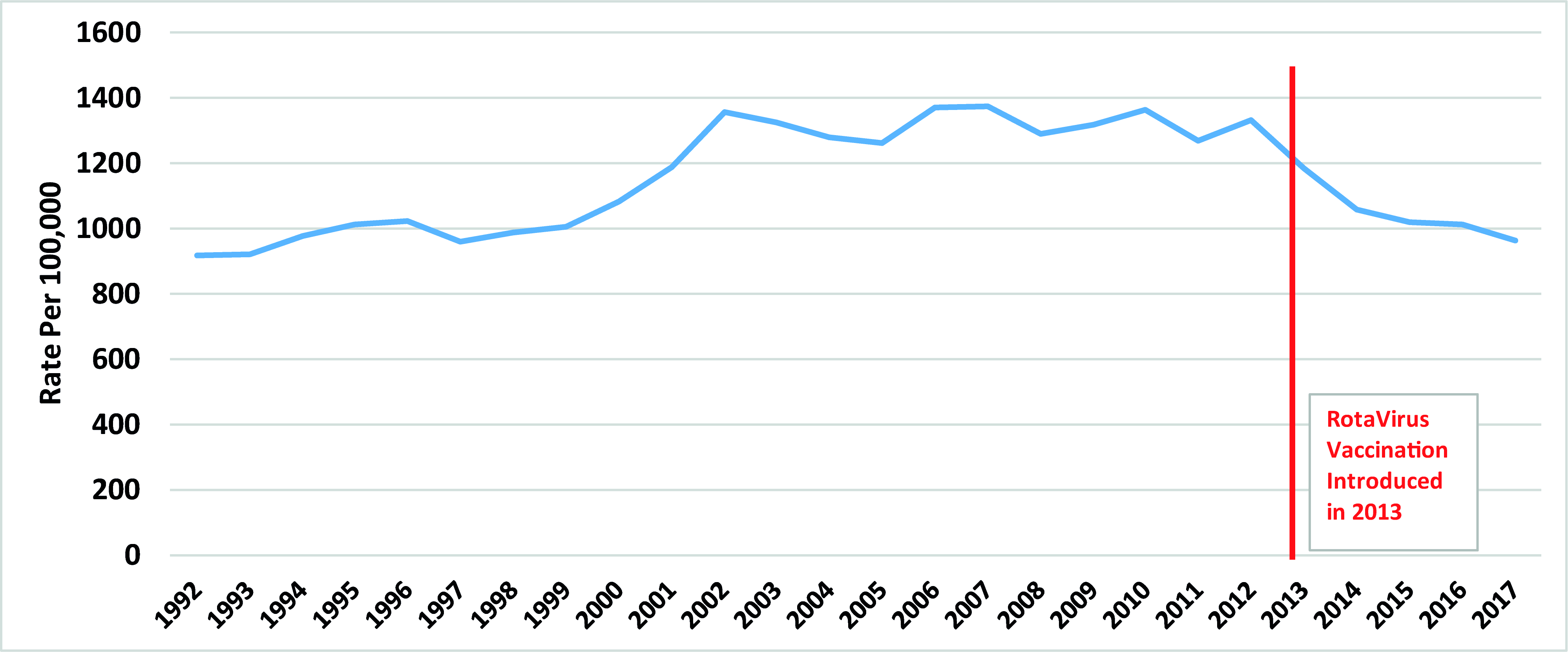

Supplement: Supplementary file 1 — Additional file 1: Figure S1. Incidence of gastroenteritis (age and gender standardised rate per 100,000 registered) in RCGP RSC sentinel practices 1992-2017 (standardised against the 2011 Census). [file 12889_2020_8525_MOESM1_ESM.tif]

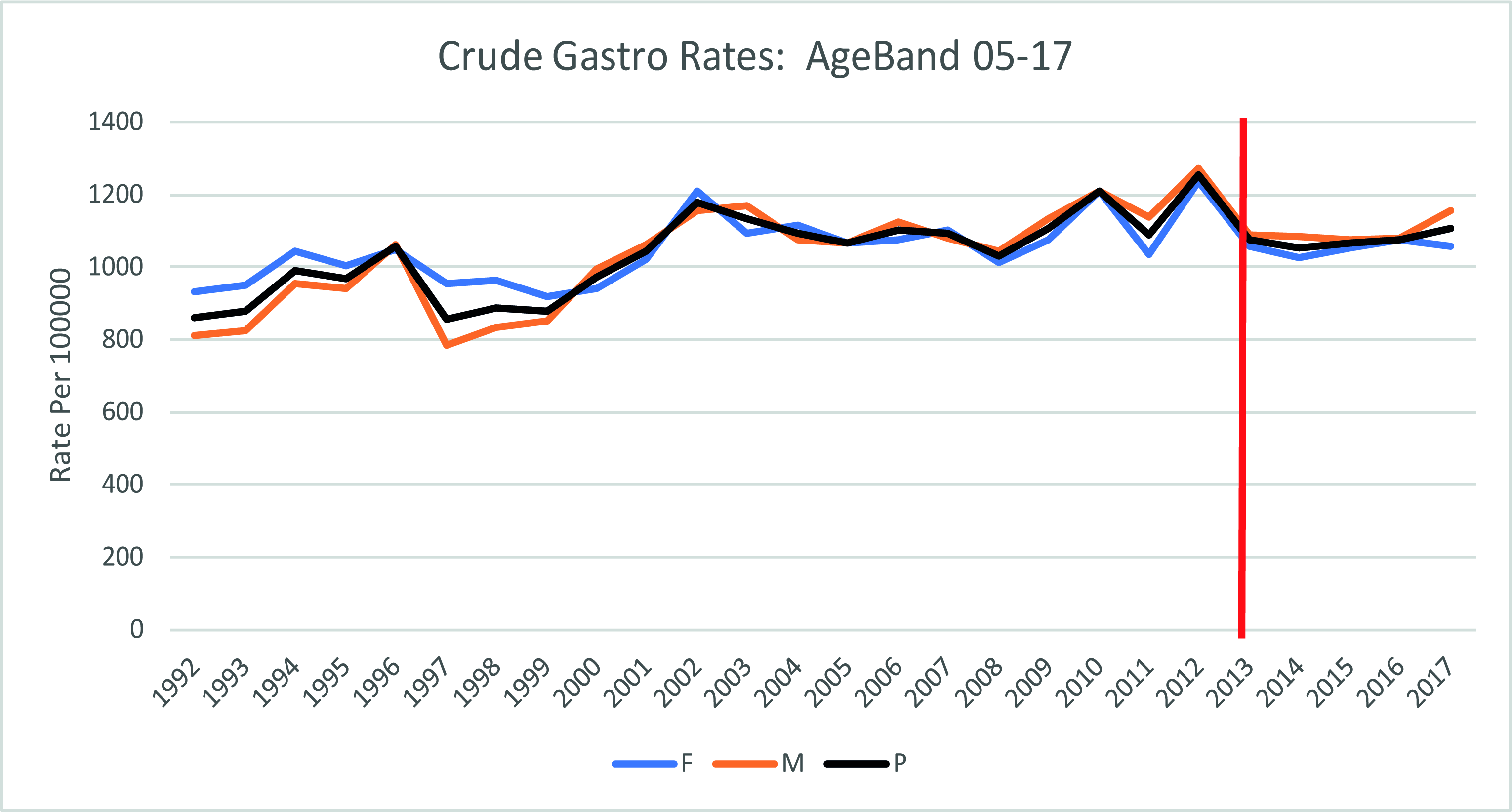

Supplement: Supplementary file 2 — Additional file 2: Figure S2. Crude gastroenteritis rates age band 05-17years. [file 12889_2020_8525_MOESM2_ESM.tif]

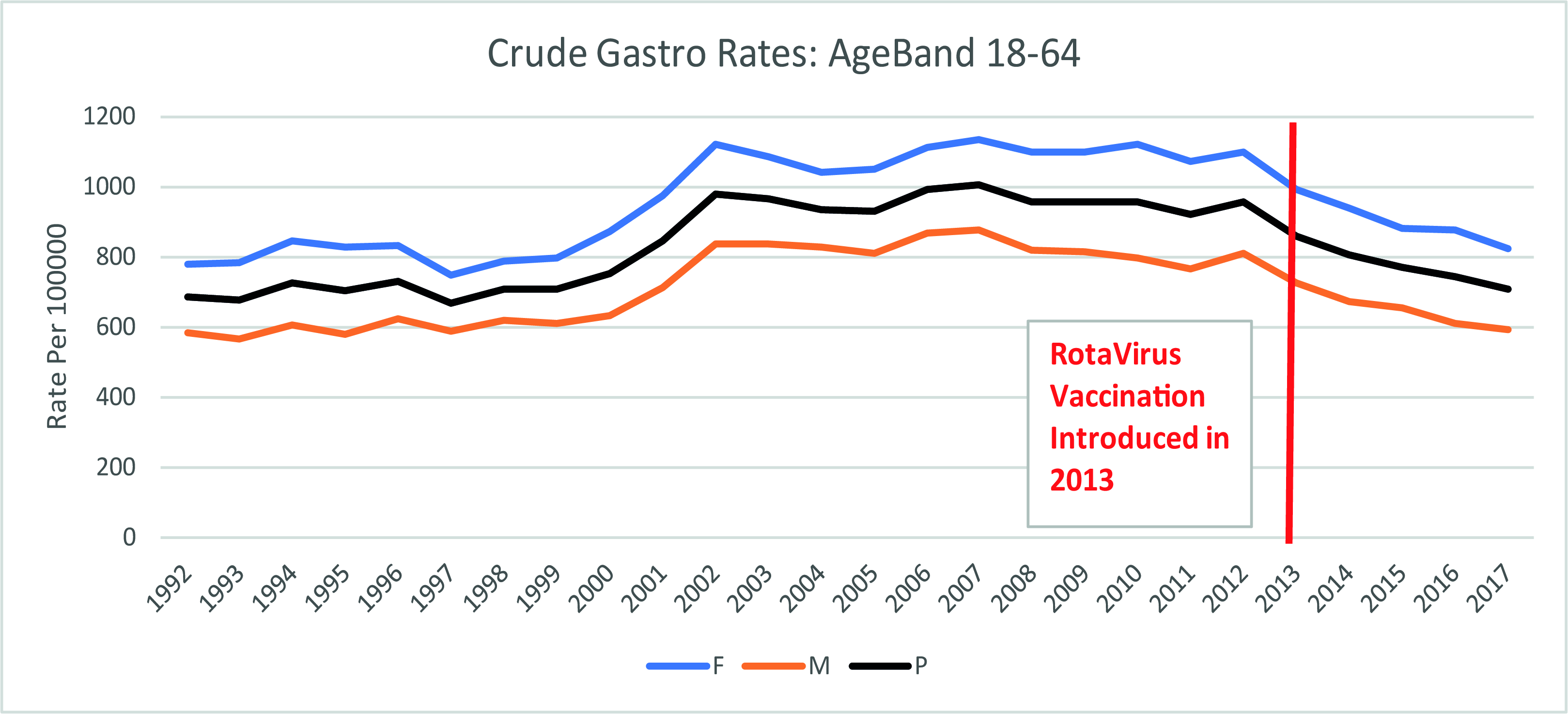

Supplement: Supplementary file 3 — Additional file 3: Figure S3. Crude gastroenteritis rates age band 18-64years. [file 12889_2020_8525_MOESM3_ESM.tif]

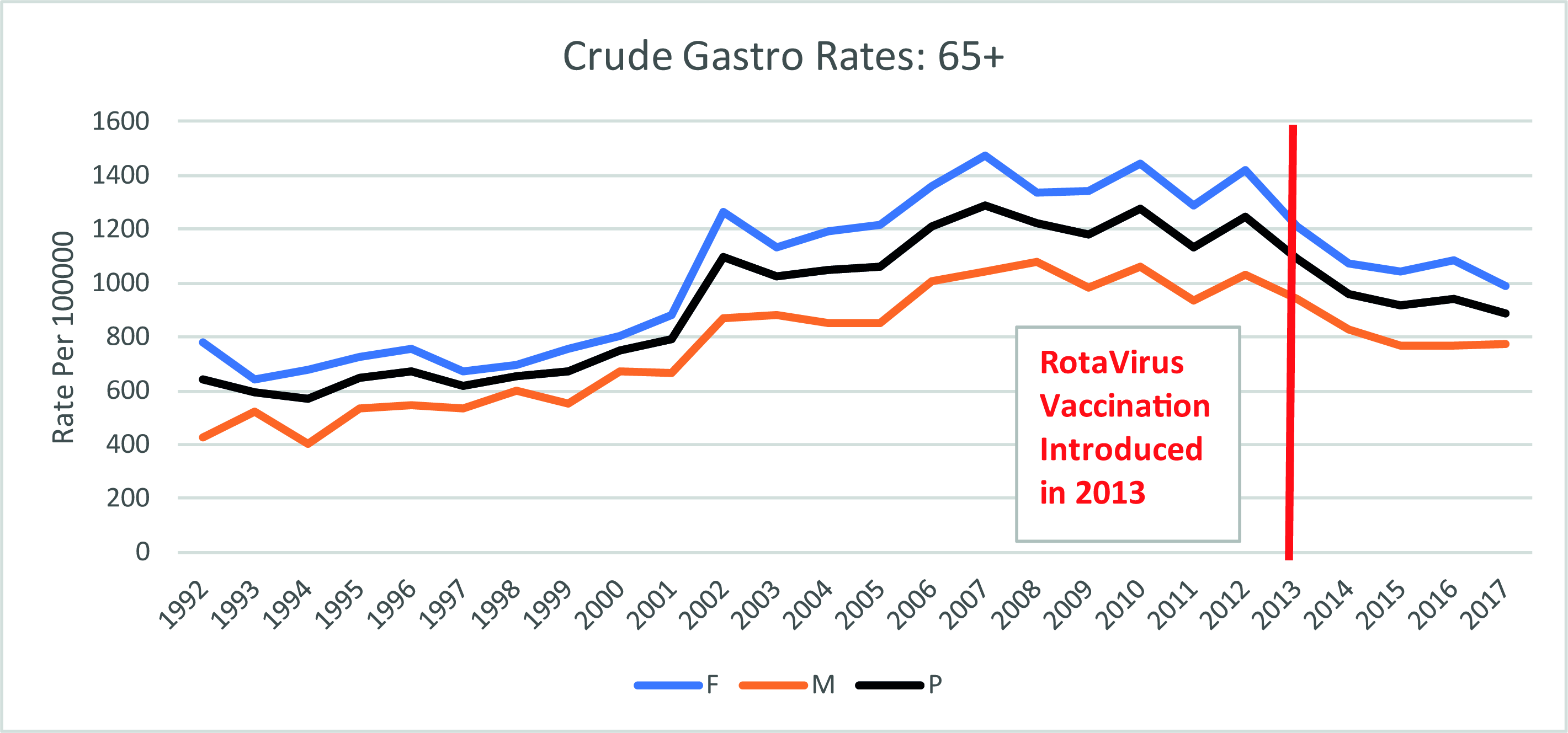

Supplement: Supplementary file 4 — Additional file 4: Figure S4. Crude gastroenteritis rates age band 65 and above. [file 12889_2020_8525_MOESM4_ESM.tif]

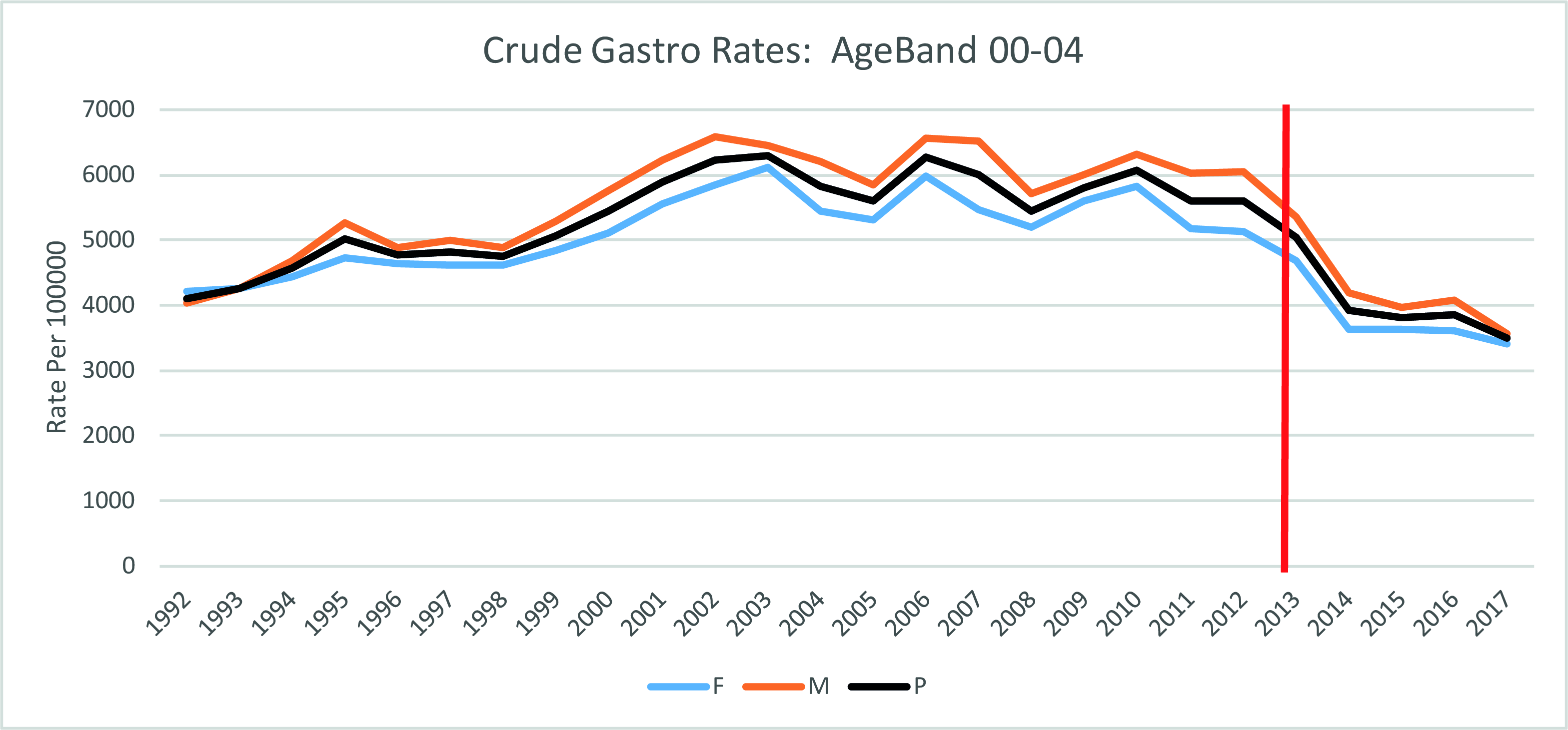

Supplement: Supplementary file 5 — Additional file 5: Figure S5. Crude gastroenteritis rates age band 00- 4years. [file 12889_2020_8525_MOESM5_ESM.tif]

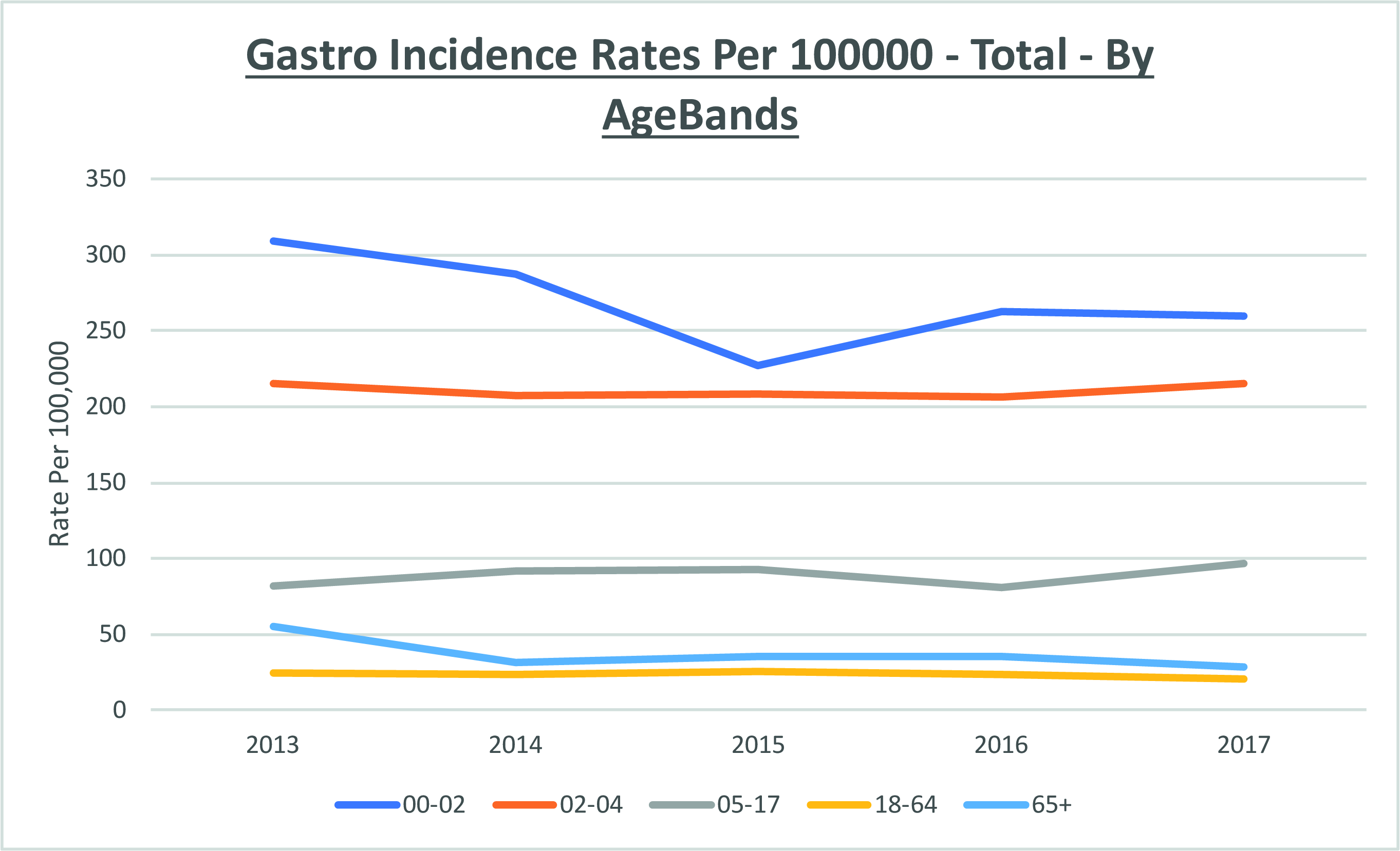

Supplement: Supplementary file 6 — Additional file 6: Figure S6. Gastro incidence rates per 1000 total by age bands. [file 12889_2020_8525_MOESM6_ESM.tif]

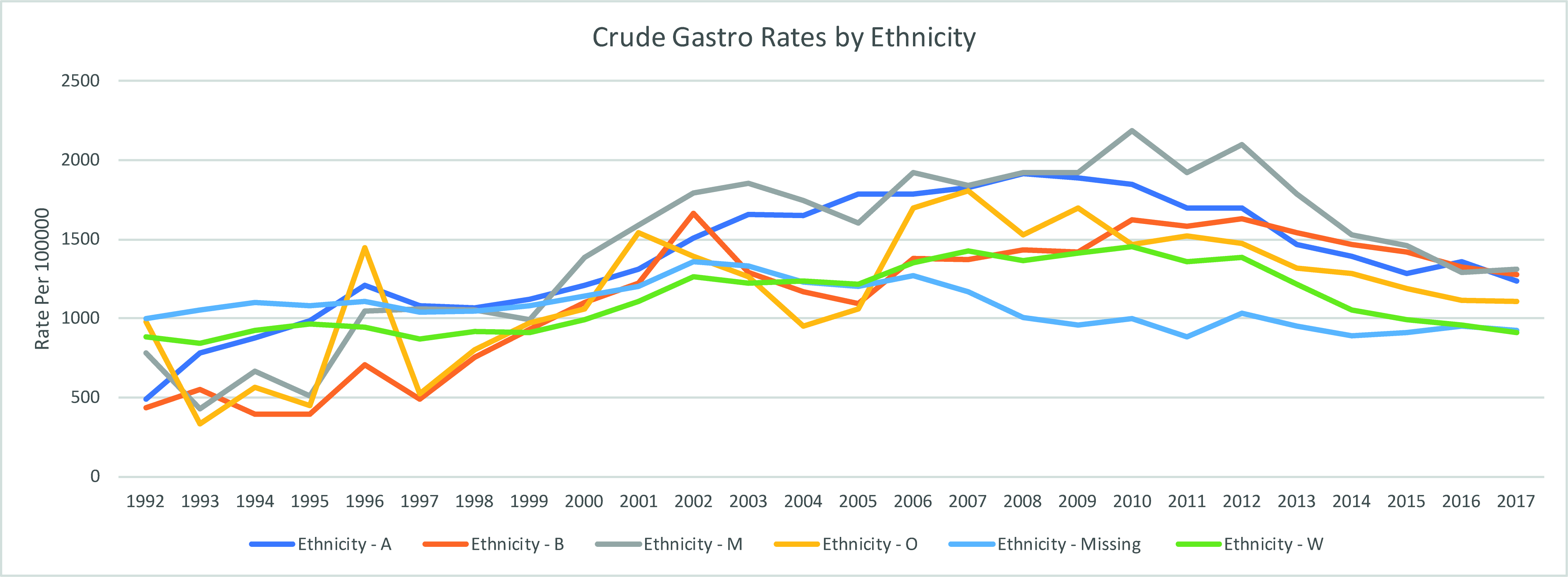

Supplement: Supplementary file 7 — Additional file 7: Figure S7. Crude gastroenteritis rates by ethnicity. [file 12889_2020_8525_MOESM7_ESM.tif]

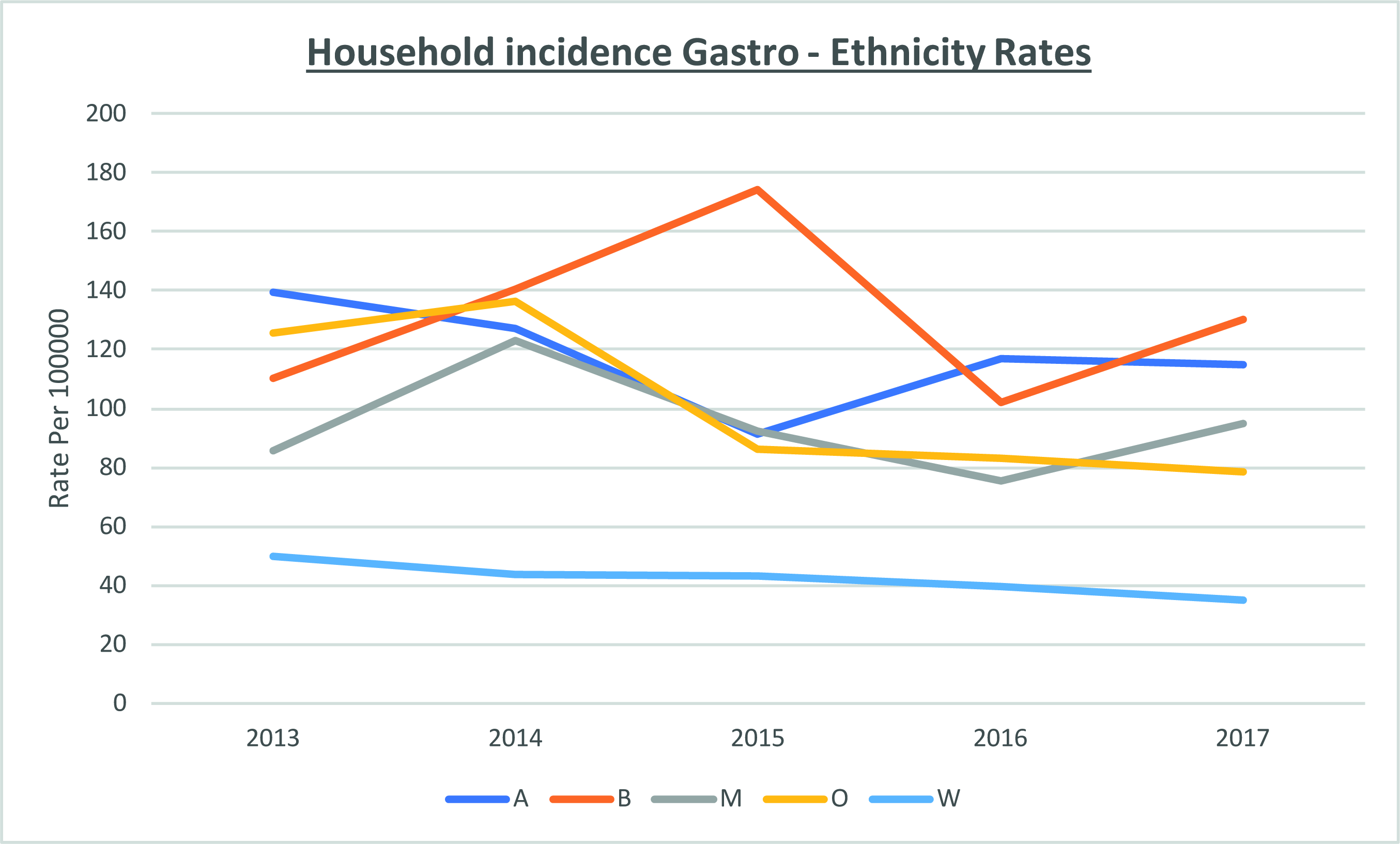

Supplement: Supplementary file 8 — Additional file 8: Figure S8. Household incidence gastroenteritis ethnicity rates. [file 12889_2020_8525_MOESM8_ESM.tif]
