# Supplementary material for: Household presentation of acute gastroenteritis in a primary care sentinel network: retrospective database studies
Source: BMC Public Health. 2020 Apr 5;20:445. doi: 10.1186/s12889-020-08525-8 (PMC7132989; doi:10.1186/s12889-020-08525-8)
Supplement: Supplementary file 17 — Additional file 17: Table S9. Variance inflationary factors associated with the Frailty Survival Model. [file 12889_2020_8525_MOESM17_ESM.docx]

S9 Table: Variance inflationary factors associated with the Frailty Survival Model

| Variables | Gvif | Df | **Gvif^(1/2*Df)** |
| --- | --- | --- | --- |
| Under 5 years old in Household | 1.215870 | 1 | 1.103 |
| Household size | 1.264194 | 1 | 1.124 |
| IMD Quintile | **1.208879** | 1 | 1.00 |
| Sex | 1.004691 | 1 | 1.00 |
| Ethnicity code | 1.424507 | 5 | 1.036 |
| Rural urban classification | 3.073544 | 2 | 1.324 |
| NHS Region | 3.371996 | 3 | 1.725 |

Vif: variance inflationary factor, Df: degree of freedom
